# Validity and Reliability of the Strengths and Difficulties Questionnaire in 5–6 Year Olds: Differences by Gender or by Parental Education?

**DOI:** 10.1371/journal.pone.0036805

**Published:** 2012-05-18

**Authors:** Cathelijne Mieloo, Hein Raat, Floor van Oort, Floor Bevaart, Ineke Vogel, Marianne Donker, Wilma Jansen

**Affiliations:** 1 Department of Youth Policy, Rotterdam Municipal Health Service (GGD Rotterdam-Rijnmond), Rotterdam, The Netherlands; 2 Department of Public Health, Erasmus MC-University Medical Centre Rotterdam, Rotterdam, The Netherlands; 3 Department of Child and Adolescent Psychiatry, Erasmus MC- University Medical Centre Rotterdam, Rotterdam, The Netherlands; UCL Institute of Child Health–, University College London, United Kingdom

## Abstract

**Introduction:**

The Strengths and Difficulties Questionnaire (SDQ) is a relatively short instrument developed to detect psychosocial problems in children aged 3–16 years. It addresses four dimensions: emotional problems, conduct problems, hyperactivity/inattention problems, peer problems that count up to the total difficulties score, and a fifth dimension; prosocial behaviour. The validity and reliability of the SDQ has not been fully investigated in younger age groups. Therefore, this study assesses the validity and reliability of the parent and teacher versions of the SDQ in children aged 5–6 years in the total sample, and in subgroups according to child gender and parental education level.

**Methods:**

The SDQ was administered as part of the Dutch regularly provided preventive health check for children aged 5–6 years. Parents provided information on 4750 children and teachers on 4516 children.

**Results:**

Factor analyses of the parent and teacher SDQ confirmed that the original five scales were present (parent RMSEA = 0.05; teacher RMSEA = 0.07). Interrater correlations between parents and teachers were small (ICCs of 0.21–0.44) but comparable to what is generally found for psychosocial problem assessments in children. These correlations were larger for males than for females. Cronbach’s alphas for the total difficulties score were 0.77 for the parent SDQ and 0.81 for the teacher SDQ. Four of the subscales on the parent SDQ and two of the subscales on the teacher SDQ had an alpha <0.70. Alphas were generally higher for male children and for low parental education level.

**Discussion:**

The validity and reliability of the total difficulties score of the parent and teacher SDQ are satisfactory in all groups by informant, child gender, and parental education level. Our results support the use of the SDQ in younger age groups. However, some subscales are less reliable and we recommend only to use the total difficulties score for screening purposes.

## Introduction

Early detection and treatment of emotional and behavioural problems in childhood may lead to considerable benefits regarding child development, wellbeing, and health [1]. To detect these problems, valid and reliable screening instruments are needed.

The Strengths and Difficulties Questionnaire (SDQ) is a relatively short instrument developed to screen for emotional and behavioural problems in children aged 3–16 years [2]. The SDQ is a 25-item questionnaire with three response categories from zero to two (not true, somewhat true, and certainly true). Of all 25 items, 15 are negatively phrased and 10 are positively phrased. The questionnaire has five subscales of five items each: emotional problems, conduct problems, hyperactivity/inattention problems, peer problems, and prosocial behaviour. The sum of the first four subscales provides a total difficulties score; a high score being less favourable. The prosocial scale provides information on protective factors of the child; a low score is less favourable. The items and scores are shown in the supporting table S1. Versions of the SDQ are available for parents and teachers, and children aged 11–16 years can complete an almost identical version. To facilitate proper screening by the preventive health care a short, easy to use, and validated instrument is needed.

The SDQ has been applied and evaluated in many countries, and seems to be a suitable instrument to detect emotional and behavioural problems in secondary school aged children [3]. Although the SDQ was developed for children aged 3 years and older, few evaluations have been made in children under 7 years of age [4]–[10]. Because different phases of a child’s development coincide with age-specific problem behaviour [11], some items in the SDQ might be less applicable or more difficult to interpret in younger children.

Most studies targeted at young children explored the factor structure of either the parent or the teacher version of the SDQ. Five and three factor solutions have been reported [7]–[8], [12]. Furthermore, different patterns were found for item loadings by gender [7]–[8] but not for item loadings by parental education level [8]. Although the factor structure was invariant between groups based on parental education level, the reliability may differ between groups. Some studies reported moderate to strong internal consistency but not for all SDQ subscales [4], [7]–[9], [12]. External validity of the parent version has shown good results [5], [10]. The interrater correlation between the parent and teacher versions of the SDQ has been investigated only once [12]. Thus, although the few studies that investigated 5–6 year old children elucidated different aspects of the validity and reliability of the SDQ [4]–[9], [12], the overall picture remains fragmented.

In order to use the SDQ as an early detection instrument in children aged 5–6 years, more data are needed on the validity and reliability of the SDQ in this age group. Therefore, the aim of this study is to determine if the SDQ is a reliable and valid instrument for detecting emotional and behavioural problems in children aged 5–6 years. Data for this study were gathered as part of a regular preventive health care check of a large population sample. The Child Behavior Check List (CBCL) and corresponding Teacher Report Form (TRF) were administered in a subsample of participants to enable comparisons between the SDQ and CBCL/TRF. The CBCL and the TRF are widely used and well validated instruments for assessing emotional and behavioural problems and both contain eight syndrome scales: Anxious/Depressed, Withdrawn/Depressed, Somatic Complaints, Psychiatric Problems, Rule-Breaking Behaviour and Aggressive Behaviour, Attention Problems, and Social Problems [13]. The scales are comparable to the SDQ scales emotional problems (CBCL/TRF scales Anxious/Depressed, Withdrawn/Depressed, Somatic Complaints), conduct problems (CBCL/TRF scales Rule-Breaking Behaviour and Aggressive Behaviour), hyperactivity/inattention problems (CBCL/TRF scales Attention Problems), and peer problems (CBCL/TRF scales Social Problems). Although, the CBCL/TRF is well validated, it has several disadvantages for use in the preventive health care setting. For example, the questionnaire is long (118 questions), it contains only negative formulated questions, and it was developed for use in a clinical setting.

To consider the SDQ as a reliable and valid instrument in young children, we hypothesize the following:

1. The original five-factor structure of the SDQ can be reproduced in a sample of parents and teachers of 5 to 6 year old children.
2. The degree of agreement between the parent and teacher report in young children is higher or comparable to what is generally found for psychosocial problem assessments in children, namely a Pearson r of 0.27 [14].
3. The internal consistency of the total difficulties score and the subscales for the parent and teacher SDQ is at least 0.7 as recommended for screening instruments intended for use in groups and individuals [15].
4. The degree of agreement of the SDQ total difficulties score and subscales with the corresponding scales of the CBCL and Teacher Report Form (TRF) is larger than 0.4 [16] and larger than for all other scales (concurrent validity). The degree of agreement of the SDQ total difficulties score and subscales with the opposite scales of the CBCL and Teacher Report Form (TRF) is zero or negative (divergent validity).
5. The validity and reliability of the parent and teacher versions of the SDQ are similar in subgroups by child gender and parental education level.

## Methods

### Ethics Statement

Non-identifiable data gathered as part of the usual governmental preventive healthcare program were used. Informed consent was obtained from parents for all questionnaires that were gathered in addition to the usual practice (CBCL and TRF). This study was approved by the Medical Ethics Committee of the Erasmus University Medical Center Rotterdam, the Netherlands. This study was conducted according to the Declaration of Helsinki code of ethics.

### Data Collection

In the Rotterdam-Rijnmond area, the SDQ is routinely administered to parents and teachers as part of the preventive health check for children in grade 2 at elementary school (5–6 year olds). This assessment is routinely provided to all children in this age group as part of the Dutch preventive child healthcare program. The Dutch preventive child healthcare program offers child immunization programs as well as screening assessments for children from 0 to 19 year olds. Screening assessments are offered at 14 stages of a child’s development. At each screening, the physical health and psychosocial health of the child are assessed by a specially trained nurse or doctor. A total of 11,987 children were eligible for the preventive health check in the school year 2008–2009. In this study, we only included children of Dutch origin to limit any cross-cultural bias as ethnic background was correlated to parental education level in the present study. In accordance with the classification system used by Statistics Netherlands, we classified a child as being Dutch when both parents were born in the Netherlands [17]. Parents provided questionnaire information on 4,750 (85%) children and teachers provided information on 4,516 (84%) children. The sample consisted of 2,808 males (51%) and 2,706 females (49%). Mean age was 5.3 (SD 0.52) years. There were no differences in child age by gender (p<0.05). Parental education level was low in 13%, middle in 36% and high in 51% of the parents. There were no differences between child gender or age by parental education level (p<0.05) (Table 1). Non-response in parents was more likely when children had an elevated score on the total difficulties score of the teacher SDQ (p<0.05, eta = 0.09). Non-response in teachers was more likely when parental education was middle to high (p<0.05, eta = 0.03).

**Table 1:** Demographic characteristics of the study population.

	Parent completed forms		Teacher completed forms	
	SDQ	CBCL	SDQ	TRF
Number (n)	4,750	397	4,516	517
Gender of child (male %)	51%	55%	51%	52%
Mean age child; years (SD)	5.3 (0.52)	5.2 (0.51)	5.3 (0.51)	5.2 (0.42)
**Parental education level** *				
Low	14%	15%	14%	8%
Middle	36%	37%	35%	19%
High	50%	48%	51%	74%

Note: SDQ = Strengths and difficulties Questionnaire; CBCL = Child Behavior Checklist; TRF = Teacher Report Form.

* see text for explanation of each level.

Parents and teacher of a sub sample of children were invited to fill out the CBCL/TRF in addition to SDQ. This sample was selected in two ways: one part consisted of a random selection of children and the other part consisted of children with an SDQ score above the 90^th^ percent cut-off (p90) of 14 on the parent report or 13 on the teacher report of the SDQ. These cut offs were based on a pilot study among children eligible for a preventive health check for children in grade 2 at elementary school in the Rotterdam-Rijnmond area. In addition to the SDQ, parents of 397 children completed the CBCL and teachers of 517 children completed the TRF. Although there were differences in child age, child gender and total difficulties score of the parent and teacher SDQ between children with and without a CBCL, the effect size was small (age η^2^ = 0.005, gender η^2^ = 0.001, and total difficulties score parent η^2^ = 0.014 and teacher η^2^ = 0.008). There were differences between children with and without a TRF for child age, level of parental education and total difficulties score of the parent SDQ, but effect sizes were small (age η^2^ = 0.014, parental education level η^2^ = 0.016 and total difficulties score η^2^ = 0.001).

### Measures

The official Dutch version of SDQ was administered to parents and scored in the standard manner [18]. SDQ items and scores are shown in supporting table S1. A sub sample of parents and teachers received the CBCL/TRF [13].

Socio-demographic characteristics included child gender, child age and educational level of the parents. Parental education level was recorded as the parent with the highest education level. This was used to divide the sample into three educational levels: low (no education, primary education, or pre-vocational education), middle (secondary or vocational education), and high (bachelor or master’s degree).

### Statistical Analyses

All analyses were performed with SPSS 19.0 (SPSS Inc. 2010). Differences between parent and teacher mean scores were analyzed with a paired-sample t-test. Differences between mean scores of males and females and subgroups by parental education level were analyzed in two separate ANOVA’s with post-hoc test Games Howell because equal variance and equal group sizes were not present.

Confirmatory factor analysis was carried out to examine the factor structure of the SDQ. We used the software package MPLUS, version 4.2 [19]. Because the measurement level of the SDQ items is ordered-categorical, the weighted least squares estimator with a mean and variance adjusted chi-square statistic (WLSMV) was used [19]. For the teacher report, the COMPLEX procedure in MPLUS was used. Because children are nested within classes within schools, the data have a multilevel structure and cannot be considered as independent. Model fit was evaluated within multiple indicators of model fit, namely the Comparative Fit Index (CFI), the Tucker-Lewis Index (TLI), and the root mean square error of approximation (RMSEA). Values of CFI above 0.95 are preferred [20] but should not be lower than 0.90 [21]. Values of RMSEA lower than 0.05 are preferable but values between 0.05 and 0.08 are indicative of fair fit [22].

Interrater agreement between parents and teachers was determined with intra-class correlations (ICC) using a two-way random effect model with absolute agreement [23] and Pearson correlations. An ICC above 0.75 was considered excellent, an ICC from 0.75 to 0.40 as moderate to good, and an ICC below 0.40 as poor [16]. Differences between correlations of all subgroups were analyzed by means of the Fisher R to Z transformation [24]. A Pearson r of 0.27 or higher is comparable to what is generally found for psychosocial problem assessments in children [14].

The internal consistency of the different SDQ scales was determined by the Cronbach’s alpha coefficient. A Cronbach’s alpha of at least 0.7 is recommended for screening instruments intended for use in groups and individuals [15]. Differences between Cronbach’s alphas of all subgroups were analyzed by calculating F-statistics [25].

Concurrent validity and divergent validity of the parent and teacher SDQ were assessed by calculating the Pearson correlation between the SDQ and CBCL and the SDQ and TRF. The hypothesis for concurrent validity was that the emotional symptoms scale of the SDQ has higher correlations with the Internalizing, Anxious/depressed, Withdrawn/depressed, and Somatic complaints scale of the CBCL and TRF than all other scales. Furthermore, a higher correlation was hypothesized between the conduct problem scale of the SDQ with the Externalizing, Rule-breaking, and Aggressive scale of the CBCL/TRF, between the hyperactivity scale of the SDQ and the Attention problem scale of the CBCL/TRF, between the peer problem scale of the SDQ and the Social problem scale of the CBCL/TRF than all other scales. Finally, a high correlation was hypothesized between the total scales score of the SDQ and CBCL/TRF. For divergent validity, a negative association between the prosocial scale of the SDQ and all scales of the CBCL/TRF was hypothesized. Furthermore, a low correlation was hypothesized between the emotional symptoms of the SDQ with the externalizing subscales of the CBCL and TRF subscales, and a low correlation between the conduct problem scale, the hyperactivity scale of the SDQ, and the internalizing subscales of the CBCL/TRF scales.

All analyses were repeated separately for each subgroup by gender and by parental education level.

## Results

### Distribution of Scales

Table 2 presents mean scores and p90 cut-offs for parent and teacher ratings for the total group, by gender, and by parental education level. Teachers reported a lower level of psychosocial problems than parents for all scales did (all significant at p<0.01). Parents and teachers reported a significantly higher level of difficulties in males than in females on the total difficulties score and on four of the five subscales (p<0.05). Parents and teachers reported a significantly higher level of difficulties on the total difficulties score and on four of the five subscales in children with low parental education level than all groups by parental education level (p<0.05).

**Table 2:** Mean scores and p90 for parent and teacher SDQ by gender and parental education level.

	Total			Gender of child					Parental education level					
				Male		Female			Low		Middle		High	
		Mean (SD)	p90	Mean (SD)	p90		Mean (SD)	p90	Mean (SD)	p90	Mean (SD)	p90	Mean (SD)	p90
**SDQ parent report**		n = 4,732		n = 2,402			n = 2,323		n = 522		n = 1,414		n = 2,002	
Emotional Symptoms		1.39 (1.65)	4	1.38 (1.67)	4		1.40 (1.67)	4	1.59 (1.76)^ab^	4	1.38 (1.65)^a^	4	1.26 (1.56)^b^	4
Conduct problems		1.14 (1.36)	3	1.29 (1.47)*	3		1.00 (1.27)*	2	1.47 (1.52)^ab^	4	1.12 (1.34)^ac^	3	0.96 (1.25)^bc^	3
Hyperactivity		2.76 (2.41)	6	3.14 (2.52)*	7		2.34 (2.19)*	5	3.62 (2.59)^ab^	7	2.90 (2.42)^ac^	6	2.22 (2.16)^bc^	5
Peer problems		0.81 (1.23)	2	0.89 (1.30)*	3		0.72 (1.28)*	2	1.13 (1.47)^ab^	3	0.77 (1.15)^ac^	2	0.63 (1.07)^bc^	2
Prosocial behaviour		8.31 (1.65)	6	8.02 (1.75)*	6		8.60 (1.54)*	7	8.22 (1.76)	6	8.33 (1.63)	6	8.34 (1.62)	6
Total difficulties score		6.10 (4.58)	12	6.69 (4.88)*	14		5.48 (4.34)*	11	7.80 (5.21)^ab^	15	6.18 (4.46)^ac^	13	5.07 (3.96)^bc^	10
**SDQ teacher report**		n = 4,5101		n = 2,318			n = 2,184		n = 414		n = 1,072		n = 1,610	
Emotional Symptoms		0.97 (1.56)	3	0.94 (1.52)	3		1.00 (1.60)	3	1.26 (1.79)^ab^	4	0.93 (1.54)^a^	3	0.81 (1.43)^b^	3
Conduct problems		0.64 (1.22)	2	0.84 (1.39)*	3		0.43 (0.97)*	2	0.79 (1.31)^ab^	3	0.58 (1.09)^a^	2	0.480 (1.04)^b^	2
Hyperactivity		2.05 (2.52)	6	2.60 (2.75)*	7		1.47 (2.10)*	5	2.59 (2.73)^ab^	7	1.98 (2.46)^ac^	6	1.53 (2.18)^bc^	5
Peer problems		0.77 (1.29)	3	0.84 (1.34)*	3		0.69 (1.22)*	2	0.91 (1.42)^ab^	3	0.70 (1.25)^a^	2	0.64 (1.15)^b^	2
Prosocial behaviour		8.36 (2.07)	5	7.92 (2.27)*	5		8.83 (1.71)*	6	8.29 (2.04)^b^	5	8.43 (1.98)^c^	6	8.63 (1.90)^bc^	6
Total difficulties score		4.43 (4.54)	11	5.22 (4.88)*	12		3.59 (4.00)*	9	5.55 (5.06)^ab^	12	4.18 (4.26)^ac^	10	3.47 (3.84)^bc^	9

Note: * =  significant difference across gender p<0.05; a = significant difference between low and middle level at p<0.05; b = significant difference between low and high level at p<0.05; c = significant difference between middle and high level at p<0.05.

### Factor Structure

Confirmatory factor analyses in 4325 complete cases with parent data and 4314 complete cases with teacher data tested whether the theoretical 5-factor model of the SDQ was confirmed, namely emotional problems, conduct problems, hyperactivity/inattention problems, peer problems, and prosocial behaviour. Fit indices for the parent report approached the preferred levels (χ^2^ = 2249.57, p<0.001; CFI = 0.88; TLI = .92; and RMSEA = 0.05). Also, the fit indices for the teacher report approached the preferred levels (χ^2^ = 1402.83, p<.001; CFI = 0.89; TLI = .95; and RMSEA = 0.07) (Table 3).

**Table 3:** Goodness-of-fit indices of the SDQ by gender and by parental education level.

	χ^2^	df	p-value	CFI	TLI	RMSEA
***SDQ parent report***						
Total (n = 4,325)	2,249.57	173	<0.001	0.88	0.92	0.05
**Gender**						
Male (n = 2,192)	1,201.94	156	<0.001	0.88	0.93	0.06
Female (n = 2,094)	1,016.13	158	<0.001	0.86	0.90	0.05
**Parental education level**						
Low (n = 460)	252.60	98	<0.001	0.90	0.93	0.06
Middle (n = 1,297)	637.65	145	<0.001	0.89	0.91	0.05
High (n = 1,847)	819.50	148	<0.001	0.88	0.91	0.05
***SDQ teacher report***						
Total (n = 4314)	1,402.83	69	<0.001	0.89	0.95	0.07
**Gender**						
Male (n = 2,205)	891.06	68	<0.001	0.90	0.94	0.07
Female (n = 2,102)	635.47	64	<0.001	0.91	0.94	0.07
**Parental education level**						
Low (n = 396)	203.84	50	<0.001	0.89	0.94	0.09
Middle (n = 1,037)	307.02	55	<0.001	0.93	0.95	0.07
High (n = 1,535)	308.88	49	<0.001	0.94	0.95	0.06

Note: SDQ = Strengths and difficulties Questionnaire; df = degrees of freedom; CFI =  Comparative Fit Index; TLI =  Tucker-Lewis Index; RMSEA =  Root Mean Square Error of Approximation.

### Interrater Correlations

Interrater agreement between parent and teacher SDQ scores was determined with intra-class correlations (ICC) and Pearson correlations for all children for which a parent and a teacher report were present (n = 3,718). Correlations (ICC and Pearson) between the parent and teacher scores of complete cases in the total population were significant for all scales. The total difficulties and hyperactivity scale had an ICC ≥0.4 (p<0.001). Total difficulties score and three of the five subscales had a larger Pearson correlation than the meta-analytic mean of 0.27 [14] (Table 4).

**Table 4:** Inter-rater agreement for SDQ scores Parent x Teacher.

ICC (Pearson)	Total	Gender of child		Parental education level		
		Male	Female	Low	Middle	High
	n = 3718	n = 1913	n = 1810	n = 411	n = 1068	n = 1607
**SDQ scales**						
Emotional Symptoms	0.28 (0.29)	0.27 (0.28)	0.28 (0.29)	0.26 (0.27)	0.29 (0.30)	0.29 (0.30)
Conduct problems	0.23 (0.25)	0.25 (0.26)*	0.16 (0.20)*	0.21 (0.24)	0.20 (0.23)	0.25 (0.27)
Hyperactivity	0.42 (0.45)	0.44 (0.46)*	0.34 (0.38)*	0.43 (0.46)	0.42 (0.46)	0.38 (0.40)
Peer problems	0.29 (0.29)	0.33 (0.33)*	0.24 (0.24)*	0.26 (0.26)	0.28 (0.28)	0.28 (0.28)
Prosocial behaviour	0.21 (0.22)	0.20 (0.21)	0.15 (0.15)	0.32(0.32)^a^	0.18(0.18)^a^	0.22 (0.22)
Total difficulties score	0.41(0.41)	0.42 (0.42)*	0.35 (0.35)*	0.44 (0.44)	0.39 (0.40)	0.37 (0.37)

*Note:* All correlations significant at p<0.001; * =  significant difference across gender p<0.05; a = significant difference between low and middle level at p<0.05.

### Internal Consistency

Cronbach’s alphas were calculated for each subscale. Cronbach’s alphas for the total difficulties score and hyperactivity scale of the parent SDQ in the total population were ≥0.7. Cronbach’s alphas for total difficulties score and three of the five subscales of the teacher SDQ in the total population were ≥0.7 (Table 5). Cronbach’s alphas did not improve substantially when items were deleted in both the parent and teacher version.

**Table 5:** Internal consistency of the SDQ scales by gender and by parental education level.

Cronbach’s α	Total	Gender			Parental education level		
			Male	Female	Low	Middle	High
**SDQ parent report**	n = 4384		n = 2377	n = 2303	n = 473	n = 1320	1886
Emotional symptoms	0.61	0.63*		0.60*	0.64	0.61	0.60
Conduct problems	0.51	0.55*		0.44*	0.53	0.50	0.49
Hyperactivity	0.78	0.79*		0.75*	0.79^b^	0.77	0.75^b^
Peer problems	0.49	0.50		0.47	0.51^a^	0.40^a^	0.46^c^
Prosocial behaviour	0.63	0.64*		0.59*	0.67^b^	0.63	0.62^b^
Total difficulties score	0.77	0.79*		0.74*	0.81^ab^	0.75^a^	0.73^b^
**SDQ teacher report**	n = 4342	n = 2220		n = 2115	n = 398	n = 1041	n = 1546
Emotional Symptoms	0.71	0.70		0.72	0.75^a^	0.70^a^	0.72
Conduct problems	0.60	0.62*		0.51*	0.57	0.53	0.54
Hyperactivity	0.85	0.85*		0.81*	0.87^ab^	0.84^ac^	0.82^bc^
Peer problems	0.56	0.56		0.55	0.58^b^	0.57^c^	0.51^bc^
Prosocial behaviour	0.81	0.82*		0.76*	0.76^b^	0.79	0.80^b^
Total difficulties score	0.81	0.81*		0.79*	0.83^ab^	0.79^a^	0.77^b^

Note: * =  significant difference across gender p<0.05; a = significant difference between low and middle level at p<0.05; b = significant different between low and high level at p<0.05; c = significant difference between middle and high level at p<0.05.

### Concurrent and Divergent Validity

For all cases in which the SDQ and either the CBCL or TRF was present, concurrent and divergent validity of the parent and teacher SDQ were assessed by calculating the Pearson correlation between the SDQ and CBCL subscales and the SDQ and TRF subscales. Generally, the hypothesized pattern of correlation coefficients for concurrent and divergent validity between the parent/teacher report of the SDQ and CBCL/TRF was present. However, the emotional problems scale of the parent SDQ also had a substantial correlation with the CBCL’s thought problems subscale. The emotional symptoms scale of the teacher SDQ had a low correlation with the somatic complaints subscale of the TRF. Furthermore, the peer problem scale of both reports also showed substantial correlations with other CBCL/TRF scales than was hypothesized (Table 6).

**Table 6:** Concurrent and divergent validity between SDQ and CBCL/TRF.

	SDQ parent report (n = 344)						SDQ teacher report (n = 496)					
	Emotional	conduct	Hyperactivity	Peer	Prosocial	Total	Emotional	conduct	Hyperactivity	Peer	Prosocial	Total
**CBCL scale**												
Internalizing	**0.62**	*0.24*	*0.27*	0.33	*−0.09^c^*	0.51	**0.64**	*0.05^c^*	*0.16*	0.34	*−0.23*	0.42
Anxious depressed	**0.59**	*0.23*	*0.25*	0.29	*−0.10^c^*	0.48	**0.61**	*0.02^c^*	*0.12*	0.23	*−0.15*	0.35
Withdrawn/depressed	**0.43**	*0.27*	*0.19*	0.48	*−0.19*	0.46	**0.45**	*0.06^c^*	*0.16*	0.41	*−0.28*	0.38
Somatic complaints	**0.47**	*0.12^b^*	*0.16*	0.13b	*0.00^c^*	0.31	**0.22**	*0.07^c^*	*0.07^c^*	0.00^c^	*−0.01^c^*	0.13
Externalizing	*0.36*	**0.60**	0.47	0.38	*−0.28*	0.63	*0.09^b^*	**0.68**	0.45	0.32	*−0.44*	0.58
Rule-breaking	*0.28*	**0.54**	0.41	0.27	*−0.23*	0.52	*0.05^c^*	**0.61**	0.38	0.27	*−0.36*	0.49
Aggressive	*0.36*	**0.58**	0.47	0.39	*−0.27*	0.63	*0.10^b^*	**0.66**	0.45	0.32	*−0.43*	0.57
Social problems	0.43	0.36	0.34	**0.47**	*−0.24*	0.55	0.27	0.25	0.44	**0.43**	*−0.34*	0.54
Thought problems	0.51	0.37	0.40	0.38	*−0.17*	0.59	0.23	0.29	0.38	0.33	*−0.32*	0.47
Attention problems	0.35	0.47	**0.75**	0.31	*−0.23*	0.71	0.15	0.44	**0.76**	0.38	*−0.40*	0.72
Total	0.52	0.51	0.56	0.42	*−0.23*	**0.72**	0.32	0.51	0.64	0.46	*−0.47*	**0.76**

*Note:* Numbers printed **bold** are hypothesized to be high. Numbers printed *italic* are hypothesized to be low.

All correlations significant at p<0.001, b = significant at p<0.05; c = not significant.

### Scale Differences by Child Gender and by Parental Education Levels

#### Factor structure

When confirmatory factor analyses were performed for each group separately, the original five-factor structure of the SDQ was confirmed and fit indices approached the preferred levels in all subgroups by gender and by parental education level (Table 3).

#### Interrater correlations

The R to Z transformation showed that the ICCs for the total difficulties score and three of the four subscales were significantly higher for males than females (Table 4). In females, none of the scales had a moderate ICC and only two of the five subscales had a higher correlation than the meta-analytic mean (Table 3). The ICC for the prosocial behaviour scale was larger in low parental education compared to middle parental education level (p<0.05) but for all other scales there were no significant differences (Table 4).

#### Internal consistency

Calculation of the F-statistics between Cronbach’s alphas for males and females showed that the alphas of the SDQ parent version were higher for males than females for conduct problems, hyperactivity, prosocial behaviour, and total difficulties score (p<0.05). For the SDQ teacher version, almost all Cronbach’s alphas were higher for males than females (0.05) (Table 5). Cronbach’s alphas did not improve substantially when items were deleted.

By calculating the F-statistics between Cronbach’s alphas for low, middle, and high parental education level, it showed that alphas for peer problems and the total difficulties score of the parent SDQ were higher for low parental education than for both other groups (p<0.05). The alpha for hyperactivity of the parent SDQ was higher among low parental education level than high parental education level (p<0.05). With the exception of emotional symptoms and impact score, alphas of the teacher SDQ for low parental education were generally higher than middle or high parental education level (p<0.05) (Table 5). For all groups, alphas did not improve substantially when items were deleted.

#### Concurrent and divergent validity

When Pearson correlations between the SDQ and CBCL/TRF were calculated for each subgroup by gender and separately by parental education level, the pattern for males and females appeared to be similar to the total population. Only for females did the emotional problems scale of the parent SDQ also have substantial correlations on the externalizing scale of the CBCL (data not shown).

The pattern for subgroups by parental education level was similar to that in the total population (data not shown).

## Discussion

The present study, conducted in a community sample of Dutch children aged 5–6 years, is the first study, as we know, to investigate the psychometric properties (factor structure, interrater reliability, internal consistency, and concurrent and divergent validity) of the parent and teacher SDQ with an additional focus on differences by child gender and by parental education level. The results show that, in general, reliability and validity of the parent and teacher version of the SDQ in this age group are satisfactory, but there are concerns regarding reliability of the subscales. The reliability and validity of the teacher SDQ is better in all samples than the parent report, and both versions of the SDQ perform slightly better in males and in children of parents with a low education level.

Mean SDQ scores for males and the sub-group with low parental education level were less favourable than for all other subgroups. This is in line with other reports [4], [9], [12], [18], [26]–[30]. Furthermore, other studies found higher mean scores in younger children compared with older children [18], [26], [31]. In the present study, mean scores were also higher compared to a group of Dutch children aged 10–14 years [30]. It seems that SDQ mean total difficulties scores are slightly higher and, consequently, less favourable for younger children than for older children.

The original five factor structure of the SDQ, as hypothesized by Goodman et al. [2], was reproduced in a sample of parents and teachers of 5 to 6 year old children. This five-factor model was also confirmed when the data was split by child gender and by parental education level. This is in line with other research [8], [10]. Van Leeuwen et al. [10] also tested a three-factor solution, but this did not improve model fit. Additional analyses in our population using a three-factor solution also did not show improved model fit.

Interrater agreement was acceptable for the total difficulties score and three subscales in the total sample and in the sub samples by gender and parental education level, but not for the conduct problem and prosocial behaviour scale. This is inline with research among older children [3], [10]. It is possible that these behaviours are more difficult to observe and rate for parents, for example, because teachers see children interact more with other children in the classroom. Another explanation is that these behaviours are more influenced by the setting (e.g classroom versus at home) or that subjective norms of parents of and teachers differ more on these types of behaviour.

Internal consistency for the total difficulties score and the hyperactivity/inattention scale of the parent SDQ and teacher SDQ was acceptable. Internal consistency of the parent SDQ was not acceptable for the four other subscales. Internal consistency for the teacher SDQ was generally higher than for the parent SDQ. Only the alpha of the conduct problems and peer problems scales of the teacher SDQ was lower then 0.7. In the present study, a similar pattern was found by gender and by parental education level. Our findings are comparable to studies on older children where weighted mean alphas for almost all subscales of the parent SDQ were smaller than 0.7 and weighted mean alphas for the teacher SDQ on conduct problems and peer problems were lower than 0.7. [3]. Because the scales contain just five items, it should be kept in mind that scales with a small number of items are generally less reliable than scales with more items [32]. Another explanation for smaller reliability of the subscales is that the items are less one- dimensional than assumed. For instance, the conduct problems scale asks about aggressive behaviour as well as rule-breaking behaviour.

For all scales except the peer problems scale, concurrent and divergent validity of the parent and teacher SDQ was acceptable and implies that, as hypothesized, the SDQ scores correlate with CBCL/TRF scores. However, our data should be interpreted with caution due to the small sample sizes in the subgroups by gender and by parental education level. The concurrent validity found in this study is slightly lower than that found by Goodman et al. [5] but is similar to that found in children aged 8–16 years in the Netherlands [18] and in children aged 5–8 years in Flanders [5], [12].

Finally, there were differences in validity and reliability between subgroups by gender and by parental education level. The outcomes of reliability and validity measures of the parent and teacher SDQ are better in males than females. When analyzed by parental education level, we found better internal consistency for parents with a low education level. However, differences between gender and parental education level were small and conclusions on the acceptability of the psychometric properties stayed the same for all subgroups.

It should be acknowledged that the present study has a few shortcomings. First, among parents non-response was more likely when children had an elevated score on the total difficulties score of the teacher SDQ (p<0.05). It is possible that these children were already receiving care and the parents did not wish to participate in this study; however, the effect size was very small and did not influence our results (Eta = 0.09). Teacher non-response was higher when parental education was middle to high. Parents were allowed to raise objections about scores on the teacher report; perhaps higher educated parents are more likely to raise objections than lower educated parents. Also, higher educated parents gave their children lower total difficulties scores than low educated parents. However, the effect size was again small (Eta = 0.03). Also, because no measure was included to validate the prosocial behaviour scale, we could only investigate the divergent validity and not the concurrent validity of this positively phrased subscale. Finally, because this study did not include a retest, the test-retest reliability could not be investigated.

A strength of the study is the large sample of young children for whom parent and teacher versions of the SDQ (including the impact scale) were available. This large sample was compiled in the preventive youth healthcare setting; therefore, the questionnaires (as filled out by parents and teachers) were used in the preventive child healthcare system and were not anonymous. Theoretically, this could have caused lower or higher mean outcomes, interrater agreement, and reliability than in the case of an anonymous questionnaire. Thus, generalizing our findings to an anonymous research setting probably requires caution. Finally, our study was conducted in a sample of Dutch children only. Reviews indicate that the reliability and validity of the SDQ in Western countries is comparable [3], [33]. Although Dutch children seem to have lower mean scale scores, we expect that our results can be generalized to other young populations.

In general, reliability and validity of the total difficulties score of the SDQ were satisfactory in a population of parents and teachers of young Dutch children. Overall it seems that reliability and validity were comparable to findings in populations of older children; however, as also found in older children [3], concerns remain regarding the reliability of the subscales. Because most subscales have low internal consistency and some subscales have low interrater agreement, we recommend using only the total difficulties score for screening purposes. This means that child health professionals should only use the total difficulties score as an indicator for psychosocial problems and not the individual scores on the subscales. The subscales could be further explored in their consult with the parent and child to get an indication of the kind of problems if necessary. For epidemiological studies or outcome measures in research, we recommend only using the total difficulties score. Additionally, because of the low interrater agreement we recommend to use the parent and the teacher report in combination, because this gives a more complete picture of the child’s psychosocial well-being.

Since we found similar validity and reliability in subgroups by gender and parental education level the SDQ is suitable for large screening programs in the general population. To use the SDQ as a screening tool, cut offs are needed. For Dutch children aged 7 to 12 years old cut offs are available. As our findings indicate that mean scores for young children are higher than for older children we recommend to define separate cut offs for young children as is available for British, Australian and American children [34].

In conclusion, the validity and reliability of the total difficulties score of the parent and teacher SDQ are satisfactory in all groups by informant, child gender, and parental education level. Our results support the use of the SDQ in younger age groups. However, some subscales are less reliable and we recommend only to use the total difficulties score for screening purposes.

## Supporting Information

Table S1 **SDQ items and scores.**
(DOC)
