# Supplementary material for: Validity and Reliability of the Strengths and Difficulties Questionnaire in 5–6 Year Olds: Differences by Gender or by Parental Education?
Source: PLoS One. 2012 May 18;7(5):e36805. doi: 10.1371/journal.pone.0036805 (PMC3356337; doi:10.1371/journal.pone.0036805)
Supplement: Table S1 — SDQ items and scores. (DOC) [file pone.0036805.s001.doc]

**Table S1 SDQ scales and scale descriptions for the** parent and teacher report

| SDQ scales | Items (n) | Score range | Item description |
| --- | --- | --- | --- |
| Emotional symptoms | 5 | 0-10 | Nervous or clingy |
|  |  |  | Fears, scared |
|  |  |  | Worries |
|  |  |  | Unhappy, downhearted |
|  |  |  | Somatic complaints |
| Conduct problems | 5 | 0-10 | Fights or bullies |
|  |  |  | Lies or cheats |
|  |  |  | Steals |
|  |  |  | Tempers |
|  |  |  | Obedient |
| Hyperactivity/inattention problems | 5 | 0-10 | Distractible |
|  |  |  | Persistent |
|  |  |  | Restless or overactive |
|  |  |  | Fidgety or squirming |
|  |  |  | Reflective |
| Peer problems | 5 | 0-10 | Solitary |
|  |  |  | Best with adults |
|  |  |  | Has one good friend |
|  |  |  | Liked by others (popular) |
|  |  |  | Picked on or bullied |
| Prosocial behaviour | 5 | 0-10 | Caring |
|  |  |  | Helps out |
|  |  |  | Considerate |
|  |  |  | Shares |
|  |  |  | Kind to kids |
| Total difficulties score | 20 | 0-40 | Emotional symptoms |
|  |  |  | Conduct problems |
|  |  |  | Hyperactivity/inattention |
|  |  |  | Peer problems |
